# Supplementary material for: Metabolic and genetic risk factors associated with pre-diabetes and type 2 diabetes in Thai healthcare employees: A long-term study from the Siriraj Health (SIH) cohort study
Source: PLoS One. 2024 Jun 28;19(6):e0303085. doi: 10.1371/journal.pone.0303085 (PMC11213315; doi:10.1371/journal.pone.0303085)
Supplement: S1 Table — (DOCX) [file pone.0303085.s001.docx]

**S1 Table.** Methods for measuring laboratory parameters

| **Laboratory parameters** | **Methods** |
| --- | --- |
| **Blood parameters** |  |
| Fasting blood glucose (FBG) | Hexokinase method |
| Total cholesterol (TC)  Triglyceride (TG)  High density lipoprotein cholesterol (HDL-C)  Low density lipoprotein cholesterol (LDL-C)  Creatinine (Cr) | Enzymatic method |
| Complete blood count (CBC) | Automated analyzer model |
| Glycated hemoglobin (HbA1c) | Turbidimetric inhibition immunoassay (TINIA) |
| **Spot urine albumin-to-creatinine ratio (MAU/Cr)** |  |
| Urine albumin concentration | Immunoturbidimetric assay |
| Urine creatinine concentration | Enzymatic method |
