# Supplementary material for: Metabolic and genetic risk factors associated with pre-diabetes and type 2 diabetes in Thai healthcare employees: A long-term study from the Siriraj Health (SIH) cohort study
Source: PLoS One. 2024 Jun 28;19(6):e0303085. doi: 10.1371/journal.pone.0303085 (PMC11213315; doi:10.1371/journal.pone.0303085)
Supplement: S1 File — (DOCX) [file pone.0303085.s002.docx]

**S1 File. Genotyping analysis**

Overall, a total of 3,960 were selected for genotyping using the Illumina Asian Screening Array (ASA). ASA contained probes for genotyping a total of 659,184 SNPs. SNPs with call rate lower than 90% were excluded from further analysis, leaving 659,184 SNPs. Sample with call rate lower than 97% (N = 54) were excluded together with sample with inconsistent sex information (N = 24) inferred from genotype data versus demographic data. The detail information on how the quality controls were performed and the results were detailed in the corresponding section below. The steps taken to perform QC were outlined in S2 Fig. The commands used during the QC steps were summarized in S2 Table.


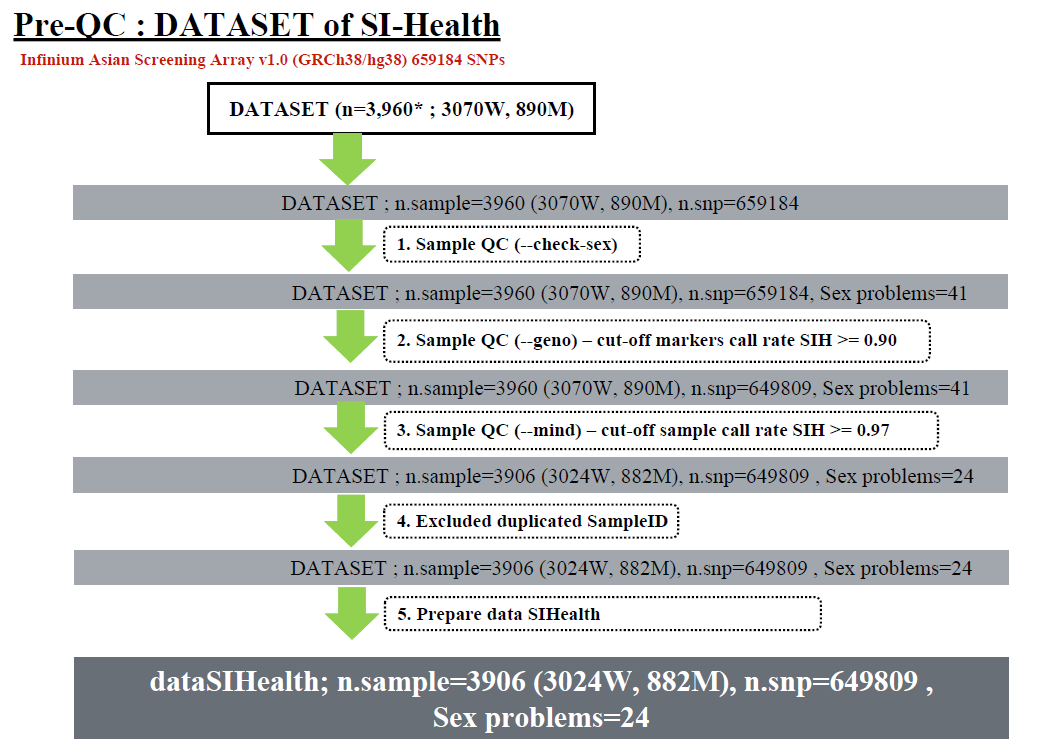


**S2 Fig.** QC Workflow for SNP Genotyping

**S2** **Table.** QC Procedures and PLINK commands used in the QC processes

| Step | Description | Input Files | Command (plink v1.9) | Output Files |
| --- | --- | --- | --- | --- |
| 1 | Check for sex inconsistency | data0 | plink --bfile data0 [command] --out data1 | data1 |
| 2 | Check both sample and marker callrate | data1 | plink --bfile data1 --missing --out projectx | projectx.imiss (individual sample missing rate) and projectx.lmiss (locus missing rate) |
| 3 | Remove markers with poor call rate | data1 | plink --bfile data1 --geno 0.1 --make-bed --out data2 | data2 |
| 4 | Recalculate sample and marker call rate | data2 | plink --bfile data2 --missing --out data2 | data2.imiss (individual sample missing rate) and data2.lmiss (locus missing rate) |
| 5 | Remove samples with poor call rate | data2 | plink --bfile data2 --mind 0.03 --make-bed --out data3 | data3 |
| 6 | Calculate MAF | data3 | plink --bfile data3 --freq --out projectx | projectx.frq |
| 7 | Calculate HWE | data3 | plink --bfile data3 --hardy --out projectx | projectx.hwe |
| 8 | Check relatedness | data3 | plink --bfile data3 --genome --out projectx | projectx.genome |

Adapt from: Turner S, Armstrong LL, Bradford Y, Carlson CS, Crawford DC, Crenshaw AT, et al. Quality Control Procedures for Genome Wide Association Studies. Curr Protoc Hum Genet. 2011 Jan;CHAPTER: Unit1.19.

Call rate for each SNP

We looked at the SNP call rate as shown in S3 Fig. The histogram showed frequency of the percentage of missing SNPs across all samples during genotyping. The median call rate was 99.65%, and the mean was 98.88% (IQR from 99.47 – 99.75). We picked the liberal call rate of 90% at this stage to keep the SNPs for future analysis.


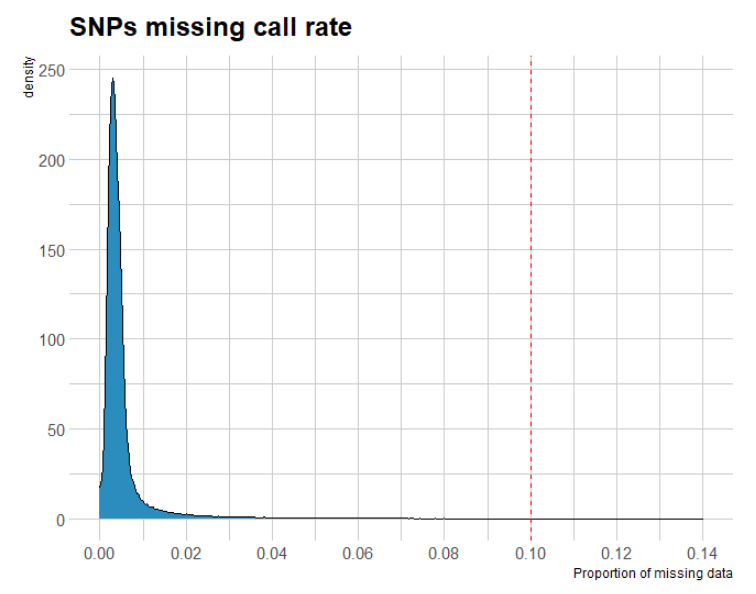

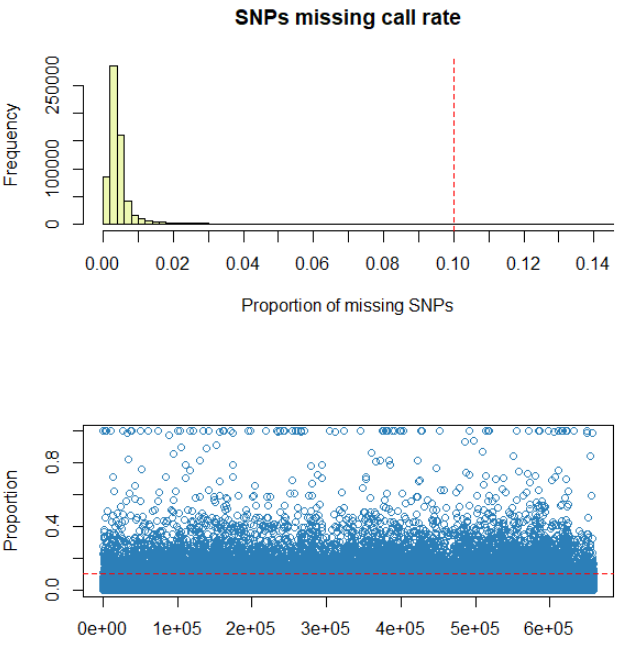


**S3 Fig.** Initial SNP call rate

# Call rate per an individual

# We looked at the SNP call rate as shown in S4 Fig. The histogram showed frequency of the percentage of missing SNPs per individual during genotyping. The median call rate was 99.24%, and the mean was 98.88% (IQR from 99.09 – 99.35). There were 73 samples with the sample call rate below 98%, 54 samples with the sample call rate less than 97%, 47 samples with the sample call rate less than 95%. We picked the liberal call rate of 97% at this stage to keep the or future analysis. Therefore, 3,906 samples were kept for further analysis. After this step, we also checked the SNP call rate again to see if any SNPs had a SNP call rate dropped below the 90% cut-off after removing these 54 samples. Since, all the SNPs still had the SNP call rate above 90%, we kept all 630,924 SNPs from the previous step.


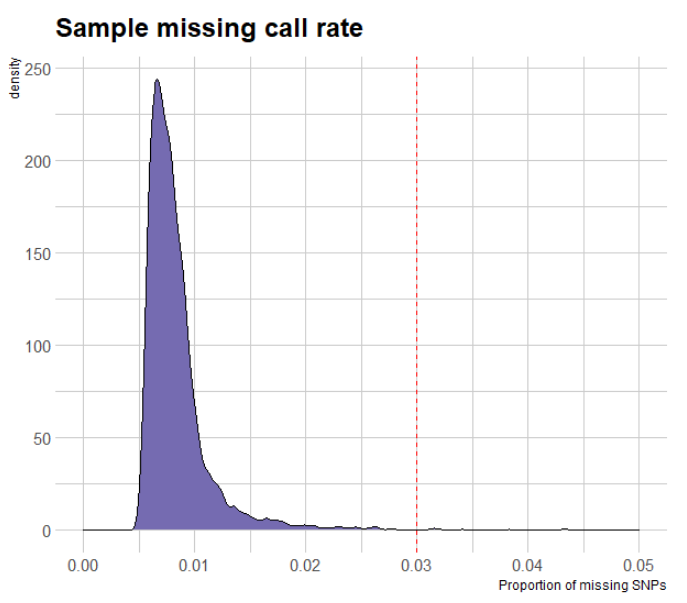

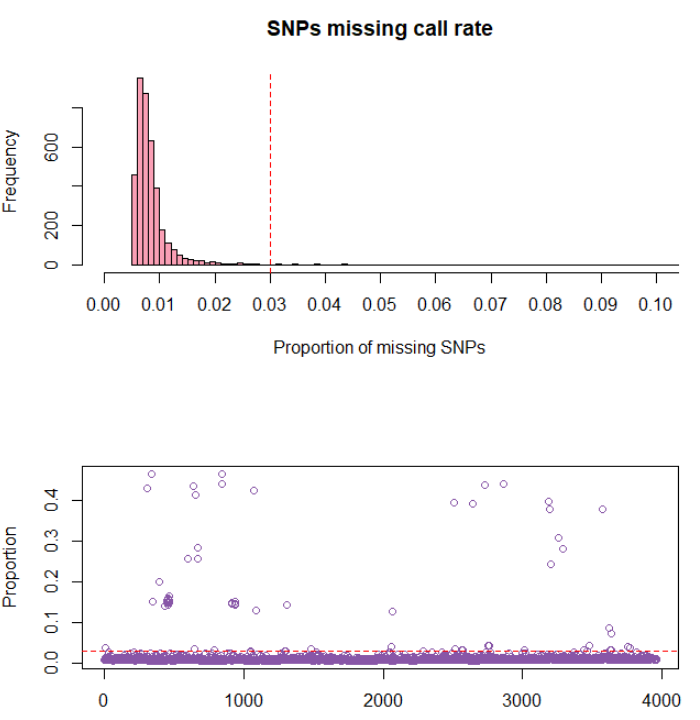


**S4 Fig.** Sample Call Rate

**SNP QC Summary**

In summary, there were 3,906 individuals and 649,809 SNPs that passed the quality control check. The detail from the above steps is summarized in S3 Table.

**S3 Table.** SNP QC Summary

| **SI Health SNP Array** | **Total Sample (cut off 97%)** | **Total SNPs (cut off 90%)** | **Sex mismatches** |
| --- | --- | --- | --- |
| Initial Data | 3,960 | 659,184 | 41 |
| After remove samples and maker poor call rate | 3,906 | 649,809 | 24 |
| After remove samples and maker poor call rate | 3,882 | 649,809 | 0 |
