# Supplementary material for: Metabolic and genetic risk factors associated with pre-diabetes and type 2 diabetes in Thai healthcare employees: A long-term study from the Siriraj Health (SIH) cohort study
Source: PLoS One. 2024 Jun 28;19(6):e0303085. doi: 10.1371/journal.pone.0303085 (PMC11213315; doi:10.1371/journal.pone.0303085)
Supplement: S2 File — (DOCX) [file pone.0303085.s003.docx]

**S2 Table.** Full model of genotypic variants of type 2 diabetes related single nucleotide polymorphisms association comparing pre-diabetes and type 2 diabetes mellitus to those without diabetes (control)

| **SNPs** | **Control** | | **Pre-DM^a^** | | **T2DM^b^** | | **Pre-DM^a^** | | **T2DM^b^** | |
| --- | --- | --- | --- | --- | --- | --- | --- | --- | --- | --- |
|  | **(n=2,671)** | | **(n=844)** | | **(n=217)** | | **OR (95%CI)** | ***P-*value^c^** | **OR (95%CI)** | ***P*-value^c^** |
| **rs7903146** |  |  |  |  |  |  |  |  |  |  |
| CC | 2439 | (91.31) | 765 | (90.64) | 195 | (89.86) | 1 |  | 1 |  |
| CT | 235 | (8.80) | 75 | (8.89) | 21 | (9.68) | 1.03 (0.63-1.69) | 0.708 | 1.07 (0.64-1.79) | 0.965 |
| TT | 8 | (0.30) | 4 | (0.47) | 1 | (0.46) | 2.74 (0.32-23.3) | 0.411 | 1.05 (0.12-9.51) | 0.972 |
| CT+TT | 243 | (9.10) | 79 | (9.36) | 22 | (10.14) | 1.07 (0.66-1.72) | 0.615 | 1.07 (0.65-1.77) | 0.791 |
| *P*-value of HWE test^d^ | 0.381 | | 0.136 | | 0.459 | |  |  |  |  |
| **rs12255372** |  |  |  |  |  |  |  |  |  |  |
| GG | 2512 | (94.05) | 788 | (93.36) | 198 | (91.24) | 1 |  | 1 |  |
| GT | 167 | (6.25) | 53 | (6.28) | 19 | (8.76) | 1.40 (0.81-2.43) | 0.250 | 1.30 (0.77-2.19) | 0.582 |
| TT | 3 | (0.11) | 3 | (0.36) | 0 | (0.00) | n/a | n/a | n/a | n/a |
| GT+TT | 170 | (6.36) | 56 | (6.64) | 19 | (8.76) | 1.33 (0.77-2.30) | 0.317 | 1.29 (0.77-2.17) | 0.342 |
| *P*-value of HWE test^d^ | 0.755 | | 0.763 | | 1.000 | |  |  |  |  |
| **rs7917983** |  |  |  |  |  |  |  |  |  |  |
| CC | 990 | (37.06) | 325 | (38.51) | 82 | (37.79) | 1 |  | 1 |  |
| CT | 1244 | (46.57) | 390 | (46.21) | 103 | (47.47) | 1.05 (0.77-1.44) | 0.572 | 1.03 (0.74-1.42) | 0.958 |
| TT | 447 | (16.74) | 128 | (15.17) | 32 | (14.75) | 0.84 (0.54-1.30) | 0.307 | 0.96 (0.61-1.52) | 0.804 |
| CT+TT | 1691 | (63.31) | 518 | (61.37) | 135 | (62.21) | 0.99 (0.74-1.34) | 0.970 | 1.01 (0.74-1.38) | 0.949 |
| *P*-value of HWE test^d^ | 0.098 | | 0.559 | | 1.000 | |  |  |  |  |
| **rs4506565** |  |  |  |  |  |  |  |  |  |  |
| AA | 2428 | (90.90) | 763 | (90.40) | 195 | (89.86) | 1 |  | 1 |  |
| AT | 235 | (8.80) | 76 | (9.00) | 20 | (9.22) | 0.97 (0.59-1.60) | 0.713 | 1.01 (0.60-1.69) | 0.999 |
| TT | 8 | (0.30) | 4 | (0.47) | 1 | (0.46) | 2.71 (0.32-23.07) | 0.411 | 1.04 (0.11-9.47) | 0.971 |
| AT+TT | 243 | (9.10) | 80 | (9.48) | 21 | (9.68) | 1.01 (0.62-1.64) | 0.981 | 1.01 (0.61-1.68) | 0.977 |
| *P*-value of HWE test^d^ | 0.381 | | 0.146 | | 0.431 | |  |  |  |  |
| **rs4132670** |  |  |  |  |  |  |  |  |  |  |
| CC | 2493 | (93.34) | 785 | (93.01) | 199 | (91.71) | 1 |  | 1 |  |
| CT | 179 | (6.70) | 55 | (6.52) | 18 | (8.29) | 1.17 (0.69-1.98) | 0.636 | 1.25 (0.71-2.21) | 0.343 |
| TT | 6 | (0.22) | 3 | (0.36) | 0 | (0.00) | n/a | n/a | n/a | n/a |
| CT+TT | 185 | (6.93) | 58 | (6.87) | 18 | (8.29) | 1.14 (0.67-1.93) | 0.629 | 1.18 (0.67-2.08) | 0.56 |
| *P*-value of HWE test^d^ | 0.151 | | 0.089 | | 1.000 | |  |  |  |  |
| **rs12243326** |  |  |  |  |  |  |  |  |  |  |
| CC | 2512 | (94.05) | 790 | (93.60) | 199 | (91.71) | 1 |  | 1 |  |
| CT | 167 | (6.25) | 51 | (6.04) | 18 | (8.29) | 1.25 (0.73-2.12) | 0.678 | 1.38 (0.78-2.42) | 0.277 |
| TT | 3 | (0.11) | 3 | (0.36) | 0 | (0.00) | n/a | n/a | n/a | n/a |
| CT+TT | 170 | (6.36) | 54 | (6.40) | 18 | (8.29) | 1.24 (0.73-2.10) | 0.438 | 1.30 (0.74-2.28) | 0.366 |
| *P*-value of HWE test^d^ | 0.756 | | 0.064 | | 1.000 | |  |  |  |  |
| **rs290487** |  |  |  |  |  |  |  |  |  |  |
| CC | 783 | (29.31) | 225 | (26.66) | 57 | (26.27) | 1 |  | 1 |  |
| CT | 1328 | (49.72) | 419 | (49.64) | 112 | (51.61) | 1.18 (0.83-1.66) | 0.699 | 1.05 (0.74-1.51) | 0.878 |
| TT | 569 | (21.30) | 197 | (23.34) | 48 | (22.12) | 1.18 (0.78-1.79) | 0.715 | 0.96 (0.62-1.47) | 0.673 |
| CT+TT | 1897 | (71.02) | 616 | (72.99) | 160 | (73.73) | 1.18 (0.85-1.63) | 0.319 | 1.02 (0.73-1.44) | 0.896 |
| *P*-value of HWE test^d^ | 0.907 | | 0.945 | | 0.684 | |  |  |  |  |

^a^Pre-DM was defined as a HbA1c level between 5.7 and 6.4%.

^b^T2DM was defined as a self-reported medical history of diabetes, current use of hypoglycemic medication, and/or a HbA1c level > 6.4%.

^c^*P*-value adjusted for age and sex; OR was an odd ratio with 95% confidence intervals (CI).

^d^HWE, Hardy-Weinberg equilibrium

SNPs, single-nucleotide polymorphisms; pre-DM, pre-diabetes; T2DM, type 2 diabetes mellitus

**S3 Table.** Association of TCF7L2 haplotype (rs7903146, rs12255372, rs7917983, rs4506565, rs4132670, rs12243326, rs290487) comparing between pre-diabetes and type 2 diabetes mellitus to those without diabetes (control)

| **No.** | **Haplotype** | **Frequency** | | | **Risk factors for Pre-DM^a^** | | **Risk factors for T2DM^b^** | |
| --- | --- | --- | --- | --- | --- | --- | --- | --- |
|  |  | **Control** | **Pre-DM^a^** | **T2DM^b^** | **OR (95%CI)** | ***P*-value^c^** | **OR (95%CI)** | ***P*-value^c^** |
| 1 | GGGGGCG | 0.3144 | 0.309 | 0.3117 | 1 | - | 1 | - |
| 2 | GGTGGCG | 0.2527 | 0.271 | 0.2694 | 1.01 (0.72-1.42) | 0.96 | 0.92 (0.67-1.27) | 0.61 |
| 3 | GGGGGCT | 0.2064 | 0.190 | 0.1956 | 0.98 (0.67-1.45) | 0.93 | 1.06 (0.73-1.52) | 0.77 |
| 4 | GGTGGCT | 0.1761 | 0.178 | 0.1702 | 1.05 (0.76-1.46) | 0.75 | 0.97 (0.71-1.32) | 0.84 |
| 5 | TTTTTTG | 0.0131 | 0.018 | 0.0184 | 1.09 (0.52-2.25) | 0.82 | 0.70 (0.27-1.79) | 0.45 |

^a^Pre-DM was defined as a HbA1c level between 5.7 and 6.4%.

^b^T2DM was defined as a self-reported medical history of diabetes, current use of hypoglycemic medication, and/or a HbA1c level > 6.4%.

^c^*P*-value adjusted for age and sex; OR was an odd ratio with 95% confidence intervals (CI).

Global haplotype association *P*-value: 0.91.

pre-DM, pre-diabetes; T2DM, type 2 diabetes mellitus

**S4 Table.** Stratified analysis of association comparing pre-diabetes and type 2 diabetes mellitus to those without diabetes (control)

| **SNPs** | **Risk allele** | **Sex** | **Risk factors for pre-DM^a^** | **Risk factors for T2DM^b^** |
| --- | --- | --- | --- | --- |
|  |  |  | **OR (95%CI)** | **OR (95%CI)** |
| rs7917983 | TT | Women | 1 | 1 |
|  |  | Men | 1.06 (0.39-2.90) | 2.81* (1.01-7.81) |
| rs290487 | TT | Women | 1 | 1 |
|  |  | Men | 0.44* (0.22-0.88) | 0.90 (0.45-1.82) |

^a^Pre-DM was defined as a HbA1c level between 5.7 and 6.4%.

^b^T2DM was defined as a self-reported medical history of diabetes, current use of hypoglycemic medication, and/or a HbA1c level > 6.4%.

**P*-value <0.001 adjusted for age; OR was an odd ratio with 95% confidence intervals (CI).

SNPs, single-nucleotide polymorphisms; pre-DM, pre-diabetes; T2DM, type 2 diabetes mellitus
