# Supplementary material for: Metabolic and genetic risk factors associated with pre-diabetes and type 2 diabetes in Thai healthcare employees: A long-term study from the Siriraj Health (SIH) cohort study
Source: PLoS One. 2024 Jun 28;19(6):e0303085. doi: 10.1371/journal.pone.0303085 (PMC11213315; doi:10.1371/journal.pone.0303085)
Supplement: S1 Fig — (DOCX) [file pone.0303085.s004.docx]

Baseline data 5,011 individuals (SIH1-2)

of Siriraj hospital, Thailand

Self-report non- DM

n = 4,612

Self-report DM

n = 209

non- DM

(HbA1c < 5.7%)

n = 3,487

unaware pre-DM (HbA1c 5.7- 6.4%)

n = 1,055

DM

(HbA1c > 6.4%)

n = 70

Excluded

Self-report uncertain DM and missing data

n = 190

Normal blood glucose levels

(FBG < 100 mg/dL)

n = 2,309

pre-DM

(FBG

100 – 124 mg/dL)

n = 120

DM

(FBG > 124 mg/dL

and/or diagnosed as DM)

n = 48

Excluded missing data from follow-up

n = 1,010

Baseline data (SIH1-2)

Follow-up data (SIH1 only)

**S1 Fig.** Diabetes classification by self-report and HbA1c among the participants (SIH1-2)
