# Supplementary material for: Metabolic and genetic risk factors associated with pre-diabetes and type 2 diabetes in Thai healthcare employees: A long-term study from the Siriraj Health (SIH) cohort study
Source: PLoS One. 2024 Jun 28;19(6):e0303085. doi: 10.1371/journal.pone.0303085 (PMC11213315; doi:10.1371/journal.pone.0303085)
Supplement: Attachment — Submitted filename: Response to Reviewers.docx [file pone.0303085.s005.docx]

Siriraj Population Health and Nutrition Research Group
Department of Research Group and Research Network
Faculty of Medicine, Siriraj Hospital,
Mahidol University

2, Wanglang Road, Siriraj,
Bangkoknoi, Bangkok,
Thailand 10700
Tel 024192834
Fax 02-4110155

February 19, 2024

Dear Editor,

We thank you for the opportunity to re-submit our revised manuscript entitled “Metabolic and genetic risk factors associated with pre-diabetes and type 2 diabetes in Thai healthcare employees: a long-term study from the Siriraj Health (SIH) Cohort Study” (Manuscript ID: PONE-D-23-40874)

The reviewers’ comments were insightful. We have responded specifically to each suggestion below. To make changes easier to be identified, I have highlighted them in yellow in the text.

The manuscript has been reviewed and approved by all of the authors. Data in this manuscript have not been previously reported/published nor is it being considered for publication elsewhere. All correspondence, editorial communications, and reprint requests should be sent to the above address.

Our responses to the editor and reviewers’ comments are below.

We strongly hope you find our manuscript suitable for publication and look forward to hearing from you.

Sincerely,

Associate Professor Korapat Mayurasakorn, MD, FRCFPT

Family Physician

Lecturer

Email: [korapat](mailto:rjd20@columbia.edu).may@mahidol.ac.th

**Response to Editor**

When submitting your revision, we need you to address these additional requirements.

**Point 1:** Please ensure that your manuscript meets PLOS ONE's style requirements, including those for file naming. The PLOS ONE style templates can be found at

<https://journals.plos.org/plosone/s/file?id=wjVg/PLOSOne_formatting_sample_main_body.pdf> and 
<https://journals.plos.org/plosone/s/file?id=ba62/PLOSOne_formatting_sample_title_authors_affiliations.pdf>

**Response 1:** Thank you for your comment. We will ensure that our manuscript meets PLOS ONE's style requirements.

**Point 2:** For studies involving third-party data, we encourage authors to share any data specific to their analyses that they can legally distribute. PLOS recognizes, however, that authors may be using third-party data they do not have the rights to share. When third-party data cannot be publicly shared, authors must provide all information necessary for interested researchers to apply to gain access to the data. (<https://journals.plos.org/plosone/s/data-availability#loc-acceptable-data-access-restrictions>)

For any third-party data that the authors cannot legally distribute, they should include the following information in their Data Availability Statement upon submission:
1) A description of the data set and the third-party source

2) If applicable, verification of permission to use the data set

3) Confirmation of whether the authors received any special privileges in accessing the data that other researchers would not have

4) All necessary contact information others would need to apply to gain access to the data

**Response 2:** Thank you for your comment. We have addressed the data availability statement as follows. The study is a part of the Siriraj Health (SIH) Cohort Study. The authors do not have the legal authority to distribute the data. However, all interested researchers can follow the respective rules and protocols of data sharing and scientific collaboration, just as the current authors have. The data are accessible upon request from the Siriraj Health study committee via email: sihealthstudy@mahidol.edu.

**Point 3:** One of the noted authors is a group or consortium "Siriraj Population Health and Nutrition Research (SPHERE) group and SIH study group". In addition to naming the author group, please list the individual authors and affiliations within this group in the acknowledgments section of your manuscript. Please also indicate clearly a lead author for this group along with a contact email address.’

**Response 3:** Thank you for your comment. We have decided to remove the “Siriraj Population Health and Nutrition Research (SPHERE) group and SIH study group" as authors, as most of members of these groups are already included as authors, and the SPHERE group is also listed in the author’s affiliations. Instead, we will acknowledge the individual members of these groups.

**Point 4:** We note that Figure 1 in your submission contain map/satellite images which may be copyrighted. All PLOS content is published under the Creative Commons Attribution License (CC BY 4.0), which means that the manuscript, images, and Supporting Information files will be freely available online, and any third party is permitted to access, download, copy, distribute, and use these materials in any way, even commercially, with proper attribution. For these reasons, we cannot publish previously copyrighted maps or satellite images created using proprietary data, such as Google software (Google Maps, Street View, and Earth). For more information, see our copyright guidelines: <http://journals.plos.org/plosone/s/licenses-and-copyright>.
We require you to either (a) present written permission from the copyright holder to publish these figures specifically under the CC BY 4.0 license, or (b) remove the figures from your submission:
a. You may seek permission from the original copyright holder of Figure 1 to publish the content specifically under the CC BY 4.0 license.

We recommend that you contact the original copyright holder with the Content Permission Form (<http://journals.plos.org/plosone/s/file?id=7c09/content-permission-form.pdf>) and the following text:

“I request permission for the open-access journal PLOS ONE to publish XXX under the Creative Commons Attribution License (CCAL) CC BY 4.0

(<http://creativecommons.org/licenses/by/4.0/>). Please be aware that this license allows unrestricted use and distribution, even commercially, by third parties. Please reply and provide explicit written permission to publish XXX under a CC BY license and complete the attached form.”

Please upload the completed Content Permission Form or other proof of granted permissions as an "Other" file with your submission.

In the figure caption of the copyrighted figure, please include the following text: “Reprinted from [ref] under a CC BY license, with permission from [name of publisher], original copyright [original copyright year].”

b. If you are unable to obtain permission from the original copyright holder to publish these figures under the CC BY 4.0 license or if the copyright holder’s requirements are incompatible with the CC BY 4.0 license, please either i) remove the figure or ii) supply a replacement figure that complies with the CC BY 4.0 license. Please check copyright information on all replacement figures and update the figure caption with source information. If applicable, please specify in the figure caption text when a figure is similar but not identical to the original image and is therefore for illustrative purposes only.
The following resources for replacing copyrighted map figures may be helpful:
USGS National Map Viewer (public domain): <http://viewer.nationalmap.gov/viewer/>
The Gateway to Astronaut Photography of Earth (public domain): <http://eol.jsc.nasa.gov/sseop/clickmap/>
Maps at the CIA (public domain): <https://www.cia.gov/library/publications/the-world-factbook/index.html> and <https://www.cia.gov/library/publications/cia-maps-publications/index.html>
NASA Earth Observatory (public domain): <http://earthobservatory.nasa.gov/>
Landsat: <http://landsat.visibleearth.nasa.gov/>
USGS EROS (Earth Resources Observatory and Science (EROS) Center) (public domain): [http://eros.usgs.gov/#](http://eros.usgs.gov/)
Natural Earth (public domain): <http://www.naturalearthdata.com/>

While revising your submission, please upload your figure files to the Preflight Analysis and Conversion Engine (PACE) digital diagnostic tool,

<https://pacev2.apexcovantage.com/>. PACE helps ensure that figures meet PLOS requirements. To use PACE, you must first register as a user. Registration is free. Then, login and navigate to the UPLOAD tab, where you will find detailed instructions on how to use the tool. If you encounter any issues or have any questions when using PACE, please email PLOS at [figures@plos.org](mailto:figures@plos.org). Please note that Supporting Information files do not need this step.

**Response 4:** Thank you for your concern. The map images in Figure 1 were created by Apinya Surawit, who is one of the authors, to use as the original images for illustrative purposes in our study only. We also uploaded the illustration file (final5.ai) that was the original file of the map images as the attached file in the other document. All of the figure files were already uploaded to the Preflight Analysis and Conversion Engine (PACE) digital diagnostic tool. And Ms. Apinya Surawit has provided a signed content permission form.

**Response to Reviewers**

**Reviewer #1**

**Point 1:** There is no new massage in this study. However, there is severe concern about the cohort sample and it seems there is sampling bias with two incoherent. A large portion of sample (4,518 of 5011) aged 18-55 and a small portion (493 of 5011) aged more than 55 years and this affect the results. These two age groups have different characteristics including increased rate of diabetes and metabolic syndrome (which is the target of this study) in the second group but all of them have been analyzed with each other that biased the results. This bias also affects the result of genotyping study because many individuals in first age group (younger group) will develop diabetes in the next ages above 50 and belong to diabetes group but currently are in normal group. The data should be re-analyzed after stratification of two samples and results should be arranged and presented after new analysis.

**Response 1:** Thank you for your feedback. We have carefully reviewed your concerns regarding potential sampling bias in our study cohort and acknowledge the importance of addressing this issue to ensure the validity of our results. We also would like to clarify that in our first enrollment, known as "Siriraj Health Study 1 (SIH-1)," we had 4,528 participants aged 18-55 years, while the second enrollment, which began in January 2020, consisted of 493 participants aged over 18 years old.

We have rephrased in Line 108-110, “Beginning in January 2020, the second phase of the Siriraj Health (SIH2) study included participants aged over 18 years with no upper age limit. However, due to the COVID-19 pandemic from 2020 to 2022, only 493 participants were enrolled.” It is worth noting that participants in both SIH1 and SIH2 did not exhibit significantly different age-group distributions, as illustrated in the following unpublished table (for your information only).

| Age, years | SIH1 (n=4,518) | | SIH2 (n=493) | | Total (n=5,011) | |
| --- | --- | --- | --- | --- | --- | --- |
|  | n | % | n | % | n | % |
| < 30 | 1,258 | 27.84 | 157 | 31.85 | 1,415 | 28.24 |
| 30 - 39 | 1,899 | 42.03 | 174 | 35.29 | 2,073 | 41.37 |
| 40 - 49 | 1,038 | 22.97 | 91 | 18.46 | 1,129 | 22.53 |
| 50 - 55 | 322 | 7.13 | 28 | 5.68 | 350 | 6.98 |
| ≥ 56 | 1 | 0.02 | 43 | 8.72 | 44 | 0.88 |

We also reanalyzed the progression of diabetes development among individuals who were not diagnosed with diabetes to evaluate the relative risk of developing pre-diabetes or diabetes at follow-up as shown in Table 4 and Fig 2.

**Reviewer #2**

**Point 1:** This manuscript addresses a very relevant subject, considering the increasing prevalence of type 2 diabetes and its complications, besides the evidence on the role of the TCF7L2 gene on the liability of developing type 2 diabetes related to some specific polymorphism, which may have differences among different populations that need clarifications. This manuscript deserves some grammar and punctuation revision. As pointed out by the authors, the lack of data related especially to HbA1C in the follow-up may have interfered with the final results of diabetes diagnosis. It reinforces the need for a more extensive and detailed study.

**Response 1:** Thank you for your feedback. We appreciate your acknowledgment of the relevance of our study topic and the importance of addressing potential genetic influences on type 2 diabetes (T2DM) susceptibility, particularly related to the TCF7L2 gene. We have explained more about TCF7L2 gene in Line 86-90, “In Asian populations, including Japanese [22], Thai [23], and Chinese [24], variants of the TCF7L2 gene, such as rs7903146, rs11196205, and rs12255372, have been identified as significant genetic risk factors for T2DM. These findings highlight the genetic heterogeneity of T2DM across different ethnic groups and underscore the importance of understanding population-specific genetic determinants of the disease.”

We acknowledge your suggestion for grammar and punctuation revision, and we will ensure to thoroughly review and enhance these aspects to improve the clarity and readability of the manuscript. Additionally, we acknowledge the importance of addressing the highlighted limitations regarding the lack of data related to HbA1C in the follow-up period due to the nature of a routine annual health examination policy, which may have influenced the final results of diabetes diagnosis, as indicated in Line 224-228, 423-424. However, we would like to clarify that although we lack HbA1C data, we do possess information related to fasting blood glucose collected during the follow-up period. This enables us to monitor the progression of diabetes development among individuals who were not diagnosed with diabetes at baseline, as illustrated in Table 4 and Fig 2.

**Reviewer #3**

**Point 1:**  Pichanun Mongkolsucharitkul et al has submitted the research study.

“Metabolic and genetic risk factors associated with pre-diabetes and type 2 diabetes in Thai healthcare 2 employees: a long-term study from the Siriraj Health (SIH) Cohort Study” for review. The study aims to explore NCD risk factors, biomarker relationships, and to develop a T2DM risk prediction model while investigating the association between T2DM and genetic variants of the TCF7L2 gene.

My review comments on the manuscript as below:

1) The chosen research question is very much relevant to the ever-increasing prevalence of Diabetes in the world.

**Response 1:** Thank you for recognizing the relevance of our chosen research question. We agree that the prevalence of diabetes globally is a critical issue, and we believe that addressing this research question will contribute to a better understanding of the challenges and potential solutions in managing this health concern. We are committed to conducting thorough and insightful research that can offer valuable insights into addressing the complexities surrounding diabetes. And we will continue to monitor the progress of NCD for this cohort.

**Point 2:**  I request the author to proofread the manuscript for grammatic errors and language (e.g line 32, 33)

**Response 2:** Thank you for your comment. We acknowledge your suggestion for grammar and language revision, and we will ensure to thoroughly review and enhance these aspects to improve the clarity and readability of the manuscript (highlighted in yellow).

**Point 3:**  Methods: Exclusion criteria and inclusion criteria may be defined more specifically.

**Response 3:** Thank you for your comment. We have added the inclusion and exclusion criteria in Line 100-104, “The inclusion criteria of the study were the Siriraj Hospital personnel who attended an annual health screening surveillance program. The exclusion criteria of the study were individuals who withheld treatment information, unable to consistently follow-up, unable to participate in this cohort in the next 2 years, or presence of contraindications for blood sampling, such as blood clotting.”

**Point 4:**  S1 Fig: Why the ‘self-reported uncertain DM’ is excluded?

**Response 4:** Thank you for your comment. The exclusion of individuals who self-reported uncertainty about whether they have diabetes, along with those with other missing data, was a decision made due to the limitations inherent in the survey data collection process. When participants are unsure about their diabetes status or when there are missing data points, it can introduce ambiguity and potential inaccuracies into the analysis. To maintain the reliability and validity of the study results, the research team opted to exclude these individuals from the analysis in this instance.

**Point 5:**  What are the criteria for metabolic syndrome, please define (line 198) how it has been coded as Yes and No

**Response 5:** Thank you for your comment. We have added the criteria for metabolic syndrome in Line 158-162, “We used the criteria of metabolic syndrome (MetS) in adults by the IDF definition with South Asian ethnic group [26]. According to the IDF definition, MetS is present if three or more of the following five criteria are met: WC ≥ 90 cm (men) or ≥ 80 cm (women), BP ≥ 130/85 mmHg, TG level ≥ 150 mg/dL, HDL-C level < 40 mg/dL (men) or < 50 mg/dL (women) and FBG ≥ 100 mg/dL.”

**Point 6:**  Research tools used, for example, the questionnaire used for participant interviews, Food frequency questionnaire is not submitted for review.

**Response 6:** Thank you for your comment. The questionnaire used for participant interviews were translated to English and uploaded as the attached file in the other documents. Food frequency questionnaire was not used for data collection during 2017-2022; however, we have been using the food frequency questionnaire in the follow-up in late 2023-2024 (Data is currently being collected).

**Point 7:**  Line 203- please explain the limitations of follow up data collection more explicitly. For example, if the participants were physically available or not,

**Response 7:** Thank you for your insightful comments regarding the limitations of our follow-up data collection process. We acknowledge that the explanation provided regarding the limitations of follow-up data was not explicit enough. We understand the importance of explicitly addressing these limitations, and we will ensure to provide a clearer explanation in the revised manuscript to better elucidate the constraints we faced in collecting comprehensive follow-up data.

We have explained more in Line 224-228, “We primarily collected follow-up data through routine electronic health check-ups, limiting access to key information like HbA1c levels, underlying diseases, family history, and lifestyle factors. Due to the nature of these check-ups, we couldn't gather extra data beyond what's routinely collected in healthcare. While participants were physically present, data collection was restricted by standard procedures during these visits.”

**Point 8:**  Line 223 - T2DM with concurrent HT - data not given. Moreover, HT data could be given as systolic, diastolic range that is specifically coexist with DM.

**Response 8:** Thank you for your comment. We have provided a systolic range and a diastolic range in Table 2 according to your recommendation.

**Point 9:**  Line 225 – Micro and macroalbuminuria, by itself are not modifiable. Only the progression to renal complications can be reduced with strict glycemic and HTN control. Please explain

**Response 9:** Thank you for your comment regarding micro and macroalbuminuria, and their modifiability. We decided to reanalyze risk factors among study participants with pre-diabetes or diabetes while excluding micro and macroalbuminuria as shown in Table 3-4 and Fig.2.

**Point 10:**  Line 251 - 253: this observation may be explained more elaborately using the results of the data analysis.

**Response 10:** Thank you for your comment. We reanalyzed the data to examine the risk factors among study participants with pre-diabetes or diabetes, comparing them with those who do not have diabetes, and explaining all the new results as shown in Table 4. We have explained more in Line 314-322, “It is noteworthy that the certain risk factors associated with both the development of pre-DM and T2DM were older age, the development of MetS, the maintenance of MetS, an increase in TG, and the maintenance of elevated TG. Participants aged 45 years or older displayed an 83% increased relative risks of developing T2DM (RRR = 1.83, 95% CI: 1.10-3.05). Participants who developed MetS displayed a three-fold increased relative risk of developing T2DM (RRR = 3.10, 95% CI: 1.77-5.43), while participants with maintained MetS displayed a two-fold increased relative risk of developing T2DM (RRR = 2.00, 95% CI: 1.13-3.54). Associations with increases in TG concentrations, whether occurring or being maintained, were noticeably significant and strong among participants diagnosed with developing T2DM.”

**Point 11:**  Line 303 – repeated word- microalbuminuria

**Response 11:** Thank you for your comment. We have reanalyzed the data to examine the risk factors among study participants with pre-diabetes or diabetes in Table 3-4. This word was removed because this factor was excluded from analysis.

**Point 12:**  Genetic analysis was not reviewed

**Response 12:** Thank you for your comment. We apologize if this section was not sufficiently clear or detailed. However, we believe that this information adequately addresses the genetic aspect relevant to our study. We appreciate your feedback and will ensure to review and revise this section for clarity in the manuscript. We have explained more about genetic analysis in Line 86-90, “In Asian populations, including Japanese [22], Thai [23], and Chinese [24], variants of the TCF7L2 gene, such as rs7903146, rs11196205, and rs12255372, have been identified as significant genetic risk factors for T2DM. These findings highlight the genetic heterogeneity of T2DM across different ethnic groups and underscore the importance of understanding population-specific genetic determinants of the disease.”, and in Line 204-210, “In this study, we focused on seven SNPs of the TCF7L2 gene, including rs7903146 (C/T), rs12255372 (G/T), rs7917983 (C/T), rs4506565 (A/T), rs4132670 (C/T), rs12243326 (C/T), and rs290487 (C/T). Genotype frequencies were tested for Hardy-Weinberg equilibrium (HWE) using the HWE Institute of Human Genetics calculator (https://ihg.gsf.de/ihg/index_engl.html) for both cases and controls through the Pearson χ^2^ test. All SNPs were analyzed using a codominant, dominant, and recessive model to examine the relationships between the two groups (T2DM and non-DM) using the “SNPassoc” package of the R version 3.3.1 (R Foundation, Vienna, Austria).”
