# Supplementary material for: Metabolic and genetic risk factors associated with pre-diabetes and type 2 diabetes in Thai healthcare employees: A long-term study from the Siriraj Health (SIH) cohort study
Source: PLoS One. 2024 Jun 28;19(6):e0303085. doi: 10.1371/journal.pone.0303085 (PMC11213315; doi:10.1371/journal.pone.0303085)
Supplement: Attachment — Submitted filename: Response to Reviewers.docx [file pone.0303085.s006.docx]

Siriraj Population Health and Nutrition Research Group
Department of Research Group and Research Network
Faculty of Medicine, Siriraj Hospital,
Mahidol University

2, Wanglang Road, Siriraj,
Bangkoknoi, Bangkok,
Thailand 10700
Tel 024192834
Fax 02-4110155

March 28, 2024

Dear Editor,

We thank you for the opportunity to re-submit our revised manuscript entitled “Metabolic and genetic risk factors associated with pre-diabetes and type 2 diabetes in Thai healthcare employees: a long-term study from the Siriraj Health (SIH) Cohort Study” (Manuscript ID: PONE-D-23-40874R1)

The reviewers’ comments were insightful. We have responded specifically to each suggestion below. To make changes easier to be identified, I have highlighted them in yellow in the text.

The manuscript has been reviewed and approved by all of the authors. Data in this manuscript have not been previously reported/published nor is it being considered for publication elsewhere. All correspondence, editorial communications, and reprint requests should be sent to the above address.

Our responses to the editor and reviewers’ comments are below.

We strongly hope you find our manuscript suitable for publication and look forward to hearing from you.

Sincerely,

Associate Professor Korapat Mayurasakorn, MD, FRCFPT

Family Physician

Lecturer

Email: [korapat](mailto:rjd20@columbia.edu).may@mahidol.ac.th

**Response to Editor**

**Point 1:** Please review your reference list to ensure that it is complete and correct. If you have cited papers that have been retracted, please include the rationale for doing so in the manuscript text or remove these references and replace them with relevant current references. Any changes to the reference list should be mentioned in the rebuttal letter that accompanies your revised manuscript. If you need to cite a retracted article, indicate the article’s retracted status in the References list and also include a citation and full reference for the retraction notice.

**Response 1:** Thank you for your comment. We acknowledge your suggestion for reference revision, and we will ensure to thoroughly recheck and enhance these aspects to improve the correct template of the reference list (highlighted in yellow). We did not cite any retracted articles in this manuscript.

**Response to Reviewers**

**Reviewer #2**

**Point 1:** Dear Authors,

Considering Siriraj Hospital personnel are involved in an annual health screening surveillance program, and about half the population is overweight and obese, it is expected that they receive diet and exercise orientations. As you didn't check behavioral changes, fasting blood glucose may not be enough to diagnose all participants and mislead the conclusions.

You spent much of your manuscript discussing the well-known risk factors for developing diabetes. I expected some comments on the possible influence of such metabolic factors on the outcomes you may have found in your genetic findings and comparisons of different age groups.

**Response 1:** Thank you for your insightful comments and suggestions regarding our manuscript. We appreciate your observation regarding the importance of assessing behavioral changes alongside metabolic markers in our study population. We agree that incorporating such assessments would provide a more comprehensive understanding of the outcomes.

Regarding the involvement of Siriraj Hospital personnel in an annual health screening surveillance program, it's pertinent to note that while the hospital does provide health check-up reports to its workers, there's no mandatory provision of dietary and exercise programs. Consequently, behavioral changes primarily occur on a voluntary basis. We will certainly consider including measures of behavioral changes in future studies to address this limitation and provide a more robust analysis.

We acknowledge that relying solely on fasting blood glucose (FBG) levels may not capture all participants at risk for diabetes. In Thailand, FBG levels serve as a standard early health screening measure, offering insights into glucose trends over time. While we did not utilize HbA1c in screening, recognizing its higher accuracy in defining diabetes, implementing more complicated tests could have resulted in increased missing data due to the logistical challenges associated with obtaining blood samples from the population.

However, it's essential to acknowledge the fundamental differences in the pathophysiology between HbA1c and fasting blood glucose. HbA1c reflects the average blood glucose levels over the past 2-3 months, providing a more stable indicator of long-term glycemic control, whereas fasting blood glucose offers a snapshot of blood sugar levels at a specific point in time. Despite these differences, in our pursuit of precise predictions, we attempted to compare HbA1c values with a limited number of samples, contrasting them with the FBG values that were tracked. Although the pathophysiology underlying HbA1c and FBG levels may differ, it was observed that the trends of both values move in the same direction, as illustrated in the following unpublished figure (for your information only).

A comparison of HbA1c and fasting blood glucose tests in general population

Furthermore, we understand your expectation for a discussion on the influence of metabolic factors on our genetic findings and comparisons across different age groups. Indeed, the observed association between the TCF7L2 gene (all 7 SNPs) and the decreased risk of type 2 diabetes mellitus (T2DM) in individuals aged 40 and above could potentially be influenced by statistical fluctuations, especially in studies with small sample sizes. This concern is particularly relevant when studying complex traits like T2DM, where genetic associations may be subtle and influenced by various environmental and genetic factors. Additionally, the researchers did not conduct a subgroup study to analyze genetic risk factors in relation to other metabolic risk factors, given the small size of the genetic sample.

As illustrated in the following unpublished table (for your information only), we observed significant associations between TCF7L2 SNPs and both pre-DM and T2DM across different age strata. Notably, the rs12243326, rs12255372, rs290487, rs4132670, rs4506565, rs7903146, and rs7917983 variants exhibited varying degrees of association with pre-DM and T2DM risk, with some associations being more pronounced in certain age groups.

| **Table** Stratified analysis of association comparing pre-diabetes and type 2 diabetes mellitus to those without diabetes (control) by age group | | | | | | |
| --- | --- | --- | --- | --- | --- | --- |
| **SNPs** | **Risk Allele** | **n of Genotype** | | **Age group** | **Risk factors for pre-DM** | **Risk factors for T2DM** |
|  |  | **Pre-DM** | **T2DM** |  | **OR (95%CI)** | **OR (95%CI)** |
| rs12243326 | G/T-T/T | 7 | 2 | < 30 |  | 1 |
|  |  | 24 | 3 | 30-39 | 0.41 (0.16-1.04) | 0.95 (0.15-5.93) |
|  |  | 23 | 13 | ≥ 40 | 0.24 (0.09-0.62)* | 0.12 (0.03-0.57)* |
| rs12255372 | G/T-T/T | 7 | 2 | < 30 |  | 1 |
|  |  | 26 | 3 | 30-39 | 0.38 (0.15-0.96)* | 0.96 (0.15-5.96) |
|  |  | 23 | 14 | ≥ 40 | 0.25 (0.10-0.66)* | 1.37 (0.42-4.48) |
| rs290487 | T/T | 26 | 4 | < 30 |  | 1 |
|  |  | 86 | 16 | 30-39 | 0.40 (0.25-0.65)* | 0.34 (0.11-1.05) |
|  |  | 84 | 28 | ≥ 40 | 0.19 (0.12-0.32)* | 0.09 (0.03-0.26)* |
| rs4132670 | G/T-T/T | 7 | 2 | < 30 |  | 1 |
|  |  | 26 | 3 | 30-39 | 0.51 (0.39-0.66)* | 0.84 (0.14-5.22) |
|  |  | 25 | 13 | ≥ 40 | 0.21 (0.16-0.28)* | 0.11 (0.02-0.51)* |
| rs4506565 | G/T-T/T | 10 | 2 | < 30 |  | 1 |
|  |  | 35 | 4 | 30-39 | 0.34 (0.14-0.84)* | 0.67 (0.12-3.76) |
|  |  | 35 | 15 | ≥ 40 | 0.20 (0.08-0.51)* | 0.10 (0.02-0.43))* |
| rs7903146 | C/T-T/T | 10 | 2 | < 30 |  | 1 |
|  |  | 35 | 4 | 30-39 | 0.39 (0.18-0.84)* | 0.67 (0.12-3.73) |
|  |  | 34 | 16 | ≥ 40 | 0.21 (0.10-0.47)* | 0.09 (0.02-0.40)* |
| rs7917983 | T/T | 19 | 4 | < 30 |  | 1 |
|  |  | 44 | 7 | 30-39 | 0.79 (0.44-1.43) | 1.06 (0.30-3.72) |
|  |  | 63 | 21 | ≥ 40 | 0.30 (0.17-0.53)* | 0.19 (0.06-0.57)* |
| **p*-value <0.001, Adjusted: Sex, BMI, WC, TC, TG, HDL, LDL | | | | | | |

Regarding the influence of metabolic factors on our genetic findings, we conducted adjusted analyses, accounting for sex, body mass index (BMI), waist circumference (WC), total cholesterol (TC), triglycerides (TG), high-density lipoprotein (HDL), and low-density lipoprotein (LDL). These adjustments allowed us to assess the independent effects of TCF7L2 SNPs on pre-DM and T2DM risk while controlling for potential confounders related to metabolic health.
